# Suppression of malignant melanoma by knocking down growth differentiation factor-15 via inhibiting PTEN/PI3K/AKT signaling pathway

**DOI:** 10.7150/jca.91892

**Published:** 2024-01-01

**Authors:** Jun Zhou, Caifeng Chen

**Affiliations:** Department of Dermatology, Shengli Clinical Medical College of Fujian Medical University, Fujian Provincial Hospital, No. 134 Dongjie Road, Fuzhou 350001, China.

**Keywords:** GDF15, malignant melanoma, PTEN, PI3K, AKT, 740Y-P

## Abstract

**Background:** Melanoma is a highly malignant tumor, and it is characterized by high mortality. Growth differentiation factor 15 (GDF15) and PTEN/PI3K/AKT signaling pathway have been proved to be related with regulation of tumors. If GDF15 could regulate melanoma through targeting PTEN/PI3K/AKT signaling pathway remain unclear.

**Methods:** EdU staining, wound healing, Transwell assay, and flow cytometry were performed to measure cell proliferation, migration, invasion, and apoptosis. GEPIA and TCGA data bases were applied to analyze the relationship between GDF15 and prognosis.

**Results:** We found that high expression of GDF15 suggested lower survival of melanoma patients, and is positively linked with advanced stage through analysis with GEPIA and TCGA data bases. Knockdown of GDF15 greatly inhibited the migration, invasion and proliferation ability of both M14 and M21 cells, but promoted cell apoptosis. However, the influence of GDF15 on M14 and M21 cells were reversed by 740Y-P, the activator of PTEN/PI3K/AKT signaling pathway. In addition, 740Y-P significantly reversed the influence of sh-GDF15 on the epithelial-mesenchymal transition (EMT) related proteins expression in M14 and M21 cell lines. Significant higher expression of GDF15 in melanoma was observed. In addition, the inhibition of PTEN/PI3K/AKT signaling pathway by knocking down GDF15 was observed in both M14 and M21 cell lines. sh-GDF15 greatly decreased the resistance of M14 and M21 to chemotherapy drugs, docetaxel and doxorubicin.

**Conclusions:** GDF15 regulated the cell proliferation, apoptosis, migration, invasion, and EMT process of M14 and M21 cell lines through targeting PTEN/PI3K/AKT signaling pathway. This research provides a novel prevention and treatment strategy for melanoma.

## 1. Introduction

Melanoma is a type of skin cancer that develops from the pigment-producing cells known as melanocytes 1. It is the most serious type of skin cancer and can spread to other parts of the body if not treated early. The incidence rate and mortality of melanoma are increasing year by year 2. Melanoma treatment typically involves a combination of surgery, radiation therapy, chemotherapy, and immunotherapy 3. However, for patients with advanced cancer, the treatment effect is poor. Seeking novel therapeutic strategy is necessary for the prevention and treatment of melanoma.

Growth differentiation factor 15 (GDF15) has been shown to be upregulated in melanoma and is associated with poor prognosis 4. It has been suggested that GDF15 may be a potential therapeutic target for melanoma 5. Several studies have demonstrated that GDF15 is involved in the regulation of cell proliferation, migration, and invasion in melanoma cells 6. In addition, GDF15 has been shown to be involved in the regulation of angiogenesis and immune evasion in melanoma 7. However, the further regulation mechanism remains unknown.

The PTEN/PI3K/AKT signaling pathway is a key pathway involved in the development and progression of many types of tumors 8. The PTEN/PI3K/AKT pathway is activated when P hosphatase and ten sin homologue (PTEN), a tumor suppressor gene, is mutated or deleted 9. This leads to increased activity of the PI3K/AKT pathway, which can promote cell proliferation, survival, and metastasis 10. Mutations in the PTEN/PI3K/AKT pathway have been found in many types of cancer, including breast, ovarian, and prostate cancer 11. In addition, the PTEN/PI3K/AKT pathway is also involved in the development of drug resistance in cancer cells 12. For example, Berberine regulated the Notch1/PTEN/PI3K/AKT/mTOR pathway and acted synergistically with 17-AAG and SAHA in SW480 colon cancer cells 10. PTEN/PI3K/Akt pathway alters sensitivity of T-cell acute lymphoblastic leukemia to L-asparaginase 13. Therefore, targeting this pathway may be a promising strategy for the treatment of cancer. We speculate that GDF15 might regulate the development of melanoma through affecting PTEN/PI3K/AKT signaling pathway.

Epithelial-mesenchymal transition (EMT) process in tumors is a process by which cancer cells undergo a transformation from epithelial cells to mesenchymal cells 14. This process is associated with increased invasiveness and metastasis of the tumor, and is thought to be a key factor in the progression of cancer 15. The EMT process is regulated by a variety of factors, including growth factors, cytokines, and transcription factors 16. If GDF15 could regulate the progression of melanoma through affecting EMT process has not been reported.

In this study, bioinformatics methods were performed to analyze the relationship between GDF15 expression and prognosis. Knockdown of GDF15 in M14 and M21 cell lines were constructed. The influence of GDF15 and 740Y-P on the cell proliferation, migration, invasion, and apoptosis were measured. We firstly demonstrated the regulatory role of GDF15 in malignant melanoma through targeting PTEN/PI3K/AKT pathway in both invitro and vivo levels. This study might provide a new understanding for the regulatory role of GDF15 in malignant melanoma.

## 2. Materials and Methods

### 2.1 Cell culture

Cell lines HEM, UACC62, A375, M14, and M21, procured from the American Type Culture Collection (ATCC, USA), were utilized. These cells were maintained at 37°C and 5% CO2 in a humidified incubator. Dulbecco's Modified Eagle Medium (DMEM, Gibco, #12491015, Langley, OK, USA) was the culture medium of choice, refreshed bi-daily. Upon achieving 70% confluence, cells were harvested for subsequent experimental applications. For treatment, 740YP was administered at a concentration of 10 μM.

### 2.2 EdU staining

Cells underwent fixation with polyformaldehyde for 15 minutes and subsequent rinsing with PBS (Gibco, #10010023) for five minutes. Incubation with Alexa Fluor 488-conjugated anti-EdU antibody (1:500, Thermo, #C10337, USA) in PBS lasted one hour. Further, cells were treated with DAPI dye (MERCK, #10236276001) for one minute, followed by a final PBS wash. A Leica Laser Scanning Confocal Microscope SP8 (Heerbrugg, Germany) facilitated observation, and Image J software quantified staining intensity.

### 2.3 Real time polymerase chain reaction (RT-PCR)

RNA extraction from cells employed TRIzol reagent (Invitrogen, #15596026, USA). Reverse transcription was executed using Takara's kit (#639537, China), and the resultant cDNA amplified via qRT-PCR (SYBR green qPCR Mix, QIAGEN, #204243, USA) on a Bio-Rad CFX96 system. Primers for GDF15 and GAPDH were used, with sequences provided. The primers are listed as follows: GDF15 (F: CTCCAGATTCCGAGAGTTGC, R: CACTTCTGGCGTGAGTATCC), GAPDH (F: GTCCATGCCATCACTGCCAC, R: AAGGCTGTGGGCAAGGTCAT).

### 2.4 Western blotting

Protein samples were isolated using RIPA buffer (Sigma, R0278, USA) with PMSF, and concentrations determined via BCA assay (Nanjing Jiancheng Bioengineering Institute, #A045-4-1, China). SDS-PAGE (10%) and subsequent wet transfer onto membranes (Milipore, GVWP02500, USA) preceded antibody incubations. Blocking utilized TBST with non-fat milk powder (Beyotime, #P0222, China), and detection was via enhanced chemiluminescence (Bio-Rad, #32106, USA). Antibodies against GDF15, β-actin, PI3K, AKT, cleaved caspase-3, Bax, N-cadherin, E-cadherin, vimentin, and GAPDH (all from abcam) were applied. GDF15 (1:1000, ab206414, abcam), β-actin (1:3000, ab5694, abcam), p-PI3K (1:2000, ab278545, abcam), PI3K (1:2000, ab302958, abcam), AKT (1:2000, ab238477, abcam), p-AKT (1:2000, ab81283, abcam), cleaved caspase-3 (1:1000, ab32042, abcam), bax (1:1000, ab32503, abcam), N-cadherin (1:2000, ab76011, abcam), E-cadherin (1:2000, ab40772, abcam), vimentin (1:1000, ab92547, abcam), GAPDH (1:3000, ab9485, abcam).

### 2.5 Transwell assay

Cell invasion was assessed in 24-well plates using Transwell inserts (BD Bioscience, #3422, USA) with an 8.0-μm pore size, coated with matrix gel (Corning, #356255). Cells were seeded in FBS-free medium in the upper chamber, and DMEM with 10% FBS in the lower chamber. After 24 hours, cells were fixed, stained with crystal violet (Sigma-Aldrich, #C0775), and quantified using a Nikon Eclipse TE300 microscope.

### 2.6 Bioinformatics analysis

GEPIA (http://gepia.cancer-pku.cn/) and TCGA (https://www.cancer.gov/about-nci/organization/ccg/research/structural-genomics/tcga) and were used to analyze role of GDF15 in survival and prognosis of melanoma patients. GEPIA and TCGA data bases were used mainly to analyze the prognosis, expression of GDF15 in melanoma tissues, the relationship between GDF15 and advanced tumor stage, and the expression of GDF15 in normal skin tissues.

### 2.7 Cell transfection

The cells were seeded in a culture dish. sh-GDF15 and sh-NC were designed and obtained from Realgene (Shanghai, China). The plasmids transfection was conducted with lipofectamine 2000 (#11668019, Invitrogen, US). The transfection effectiveness was quantified by measuring the mRNA level of GDF15 through RT-PCR after 48 h, and protein expression of GDF15 with western blotting after 96 h. Then, the transient transfection was constructed. pcDNA-GDF15 and control vectors were diluted with culture medium without serum to the final incubation concentration (50 nM).

### 2.8 Wound healing

The cells were plated and incubated for 24 h in 12-well plate (#3513, Corning). 1 mL tip was used to make straight line in the middle of well, and the distance between cells was captured. After 24 h, the distance between wound was tested again, and migrated distance was calculated with Image J software.

### 2.9 Flow cytometry

The cells were cultured as described in part 2.1, and transfected with related vectors as described in part 2.7. The cells were digested with trypsin (#108444, Sigma-Aldrich) and cells were washed with PBS (#10010023, Gibco) containing propidium iodide and Annexin V-FITC (Beyotime, #C1062L, China). The cells were incubated for 30 min in the dark, and analyzed with a flow cytometer.

### 2.10 CCK8 assay

The cells were seeded in a 96-well plate at a density of 2,000 cells per well. The cells were incubated for 24 h at 37°C in a humidified atmosphere containing 5% CO_2_. 100 μL CCK-8 reagent (Beyotime, #C0038, China) were added to each well and cells were incubated for 2 h at 37°C. The absorbance of each well at 450 nm was measured.

### 2.11 Statistical analysis

The data were represented as mean ± standard deviation. Results were statistically analyzed with SPSS (Version 18). p-value < 0.05 was set as statistical difference. T-test and ANOVA tests were applied in this research for statistical analysis.

## 3. Results

### 3.1 Increased GDF15 expression correlates with reduced survival in melanoma patients and is positively associated with advanced disease stages

**Figure 1:**
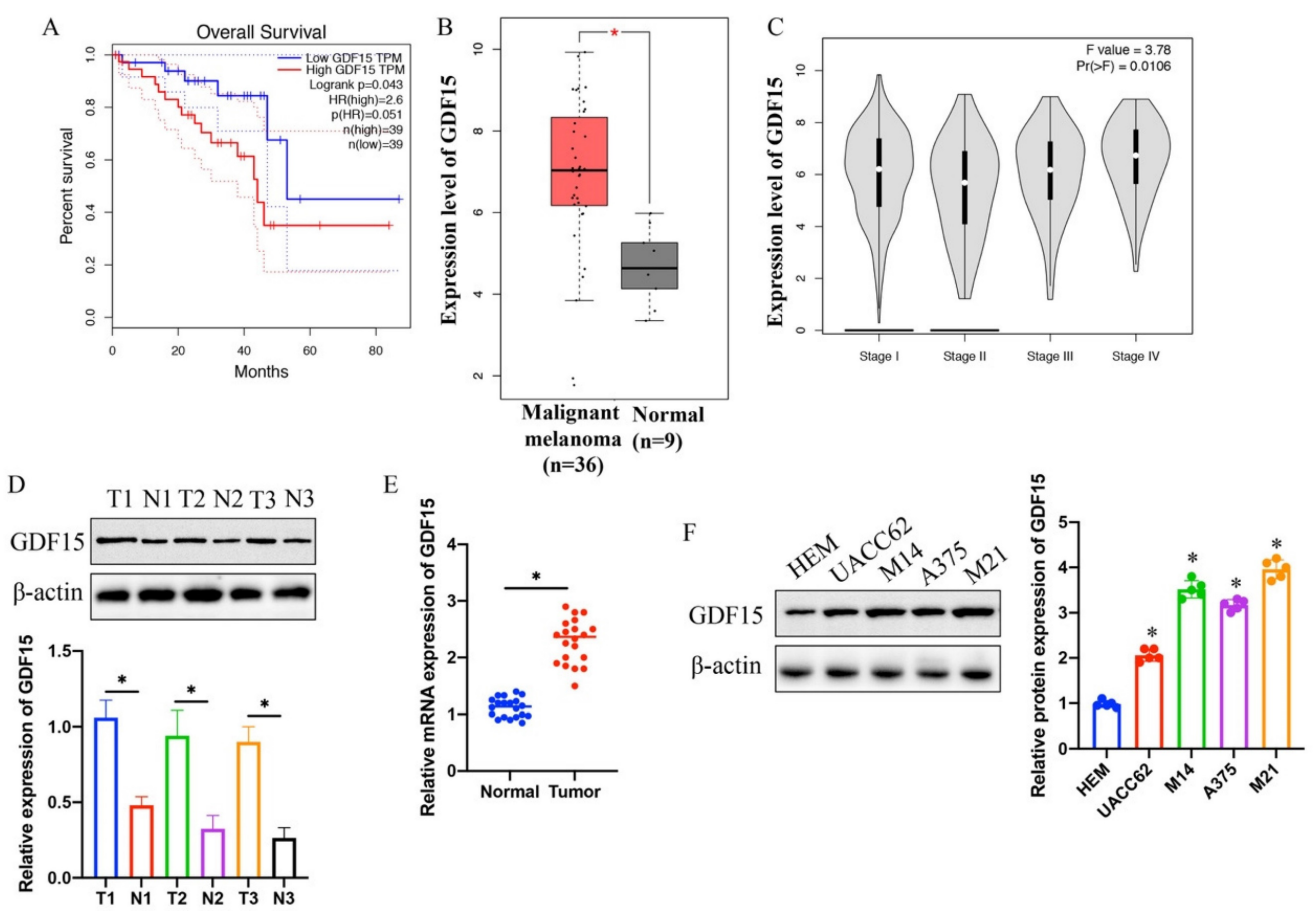
** Elevated expression of GDF15 indicates lower survival of melanoma patients, and is positively linked with advanced stage*.*** (A) Higher expression of GDF15 suggests poor prognosis of patients through analysis with GEPIA data base; (B) Significant higher protein expression of GDF15 in melanoma tissues was found through analysis with GEPIA data base; (C) Higher expression of MSN is positively correlated with advanced tumor stage; (D) The protein expression of GDF15 in melanoma tissues was greatly increased; (E) The mRNA level of GDF15 in melanoma tissues was significantly increased; (F) The protein expression of GDF15 in A375, UACC62, M14, and M21 cell lines was significantly promoted compared with group HEM cell line (n=5); * indicates p<0.05. ANOVA tests was applied in this research for statistical analysis.

Analysis using the GEPIA and TCGA databases revealed a negative correlation between GDF15 expression and survival rates in melanoma patients (Figure 1 A). A significantly higher GDF15 expression was observed in melanoma patients compared to controls (Figure 1 B). GDF15 expression levels were positively associated with tumor stage progression (Figure 1 C). Furthermore, elevated GDF15 levels were detected in melanoma tissues (Figure 1 D-E) and specific cell lines (UACC62, M14, A375, and M21) (Figure 1 F) as compared to normal skin tissues and the HEM cell line, with samples sourced from our hospital.

### 3.2 The knockdown of GDF15 substantially inhibited the PI3K/AKT signaling pathway in both M14 and M21 melanoma cell lines

**Figure 2:**
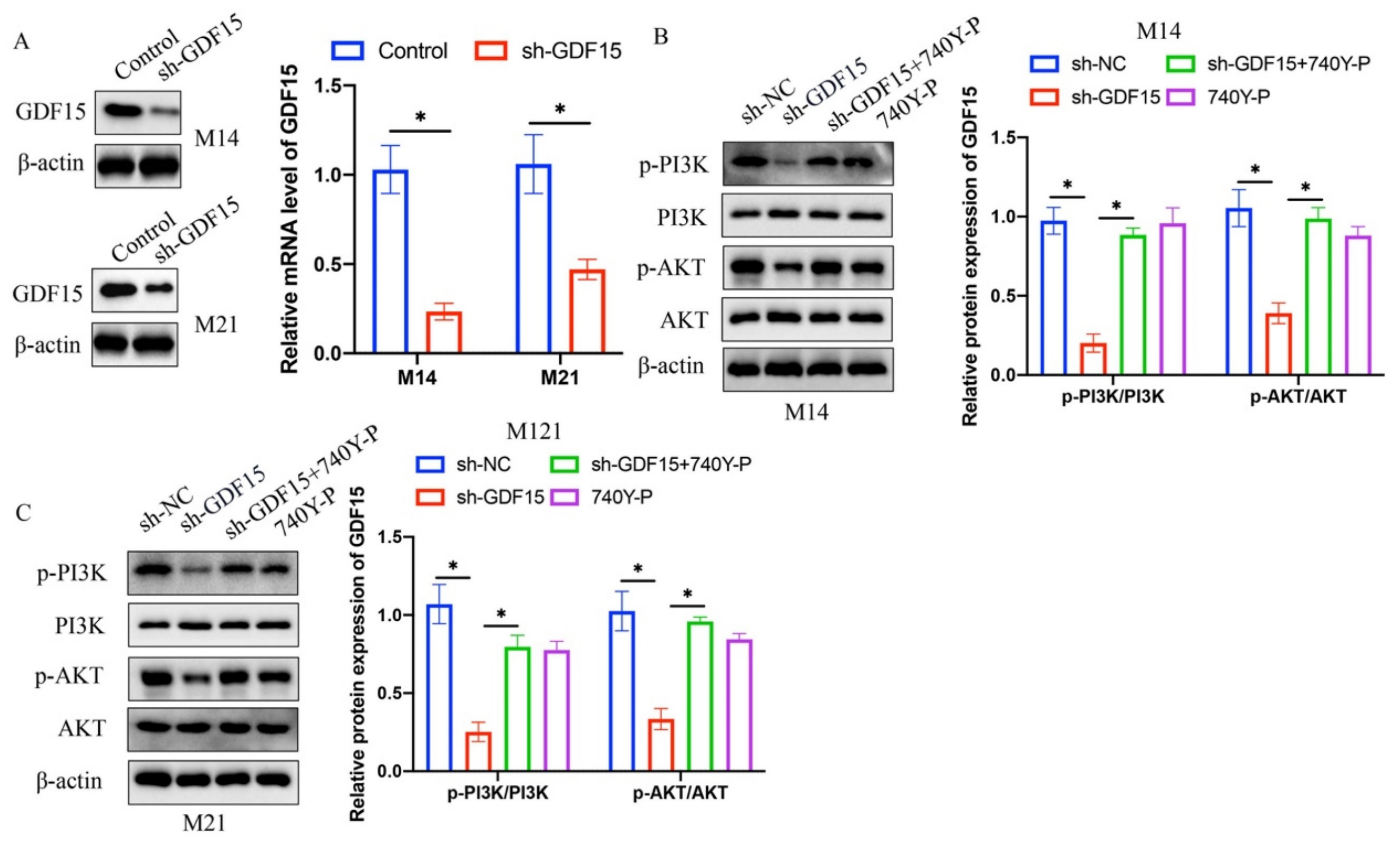
**Knockdown of GDF15 greatly suppressed PI3K/AKT signaling pathway in both M14 and M21 cell lines*.*** (A) Knockdown vectors of GDF15 were constructed in both M21 and M14 cell lines (n=3); (B) The inhibition of PI3K/AKT signaling pathway by sh-GDF15 was reversed by 740Y-P in M14 cell line (n=3); (C) The inhibition of PI3K/AKT signaling pathway by sh-GDF15 was reversed by 740Y-P in M21 cell line (n=3). * indicates p<0.05. T-test and ANOVA tests were applied in this research for statistical analysis.

Constructed knockdown vectors for miR-424-3p were successfully transfected into UACC62 and A375 cell lines (Figure 2 A). The sh-GDF15 was observed to significantly inhibit the PI3K/AKT signaling pathway in M14 and M21 cell lines (Figure 2 B-C). Subsequent experiments explored whether activation of the PTEN/PI3K/AKT pathway by 740Y-P could counteract the effects of sh-GDF15, revealing that 740Y-P notably reversed the impact of sh-GDF15 (Figure 2 B-C).

### 3.3 Concurrent treatment with 740Y-P markedly enhanced cell proliferation, migration, and invasion, which were initially diminished by sh-GDF15

**Figure 3:**
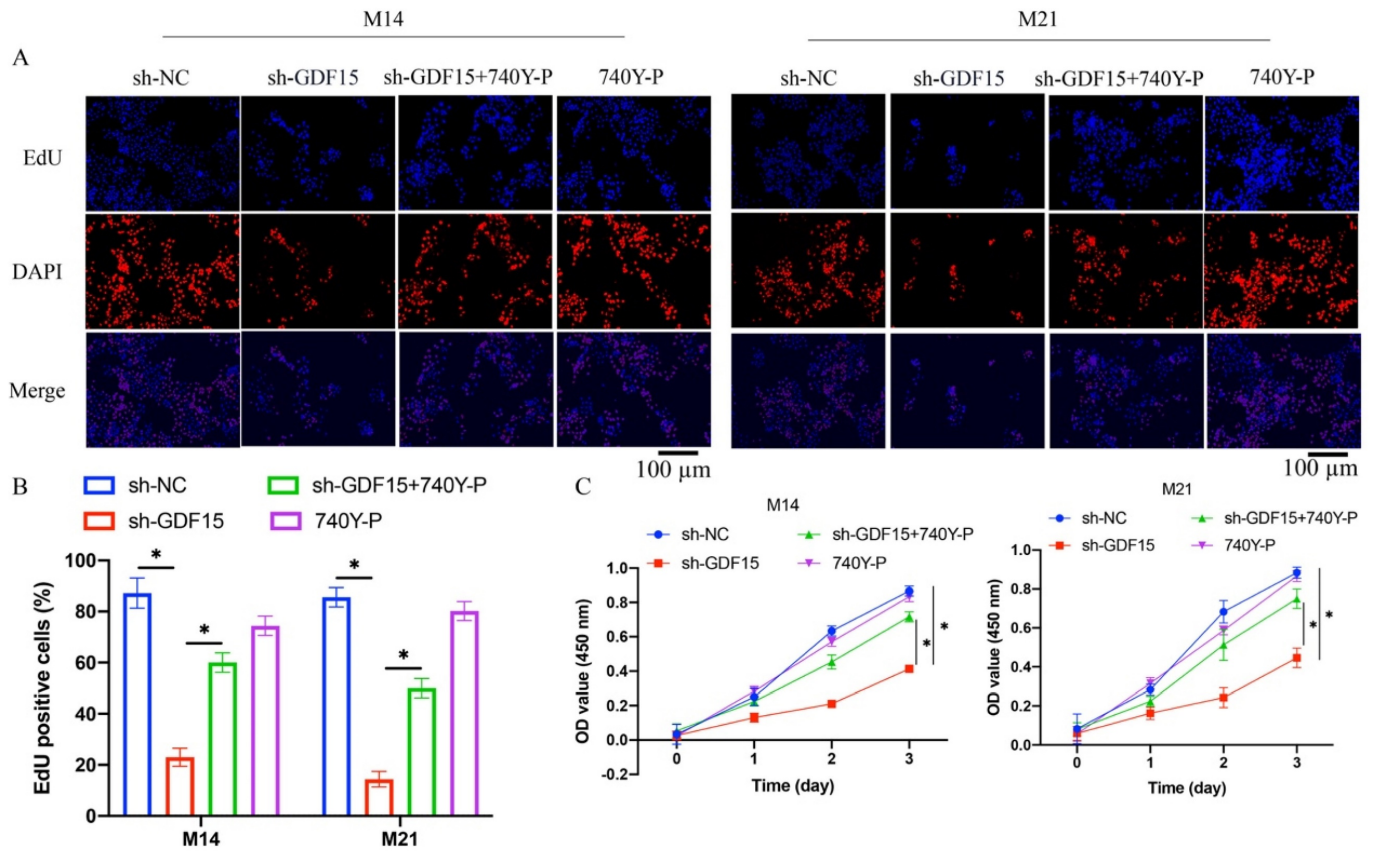
**740Y-P significantly promoted the decreased cell proliferation caused by sh-GDF15*.*** (A) The cell proliferation was investigated with EdU staining method; (B) The EdU staining data were analyzed (n=3); (C) The cell proliferation was measured with CCK8 assay (n=3). * indicates p<0.05. ANOVA tests was applied in this research for statistical analysis.

**Figure 4:**
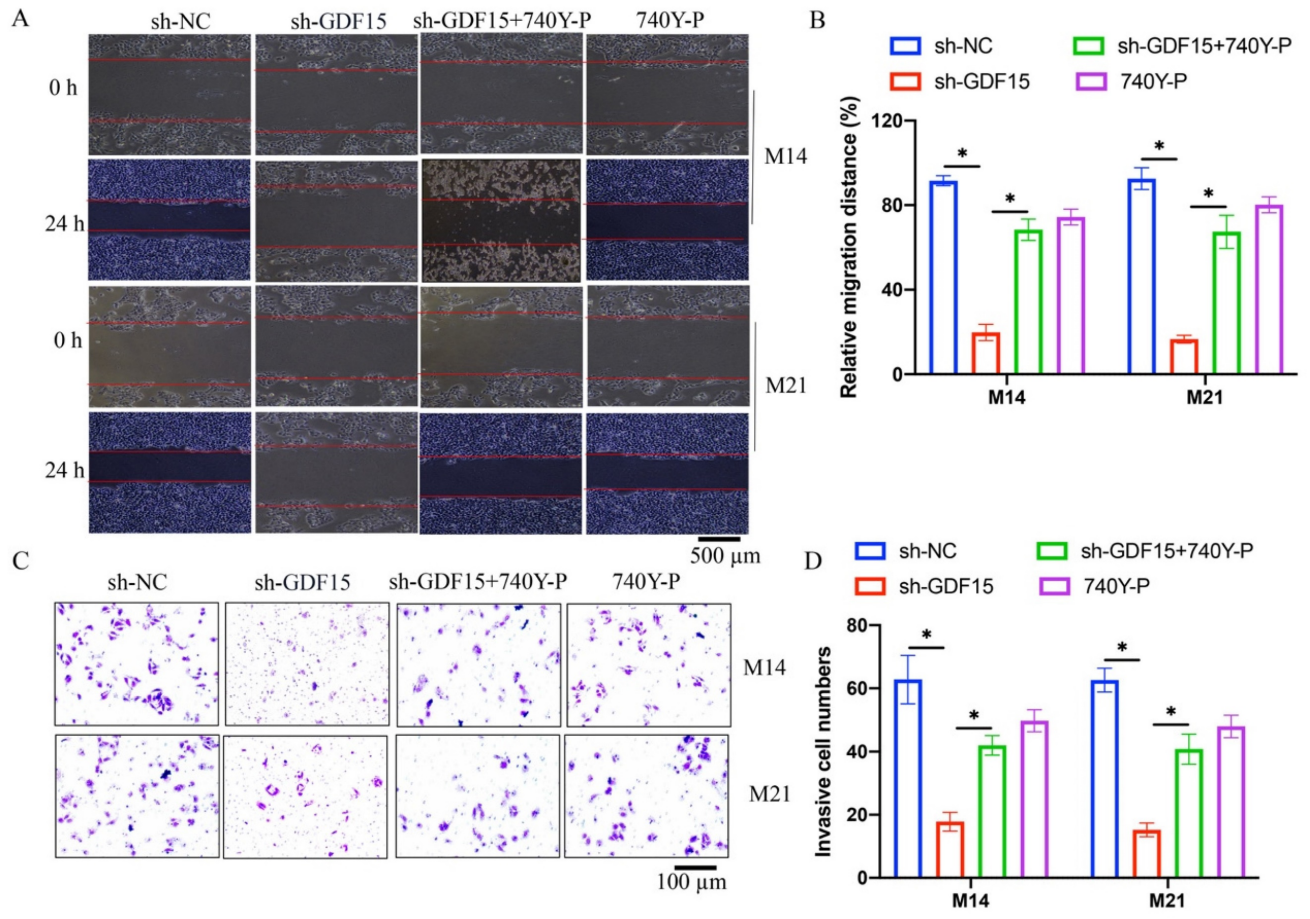
**740Y-P significantly promoted the decreased cell migration and invasion caused by sh-GDF15*.*** (A) The cell migration was investigated with wound healing method; (B) The migrated distance was analyzed (n=3); (C) The cell invasion was measured with Transwell assay; (D) The invasive cells were counted (n=3). * indicates p<0.05. ANOVA tests was applied in this research for statistical analysis.

Cell proliferation in M14 and M21 was assessed using EdU staining and CCK8 assays. sh-GDF15 markedly reduced cell proliferation (Figure 3 A-C), a decline that was reversed upon treatment with 740Y-P (Figure 3 A-C). Similarly, cell migration and invasion, assessed via wound healing and Transwell assays, respectively, were significantly inhibited by GDF15 knockdown but were restored following 740Y-P treatment (Figure 4 A-D).

### 3.4 The effects of sh-GDF15 on cell apoptosis and chemotherapy resistance were mitigated by 740Y-P

**Figure 5:**
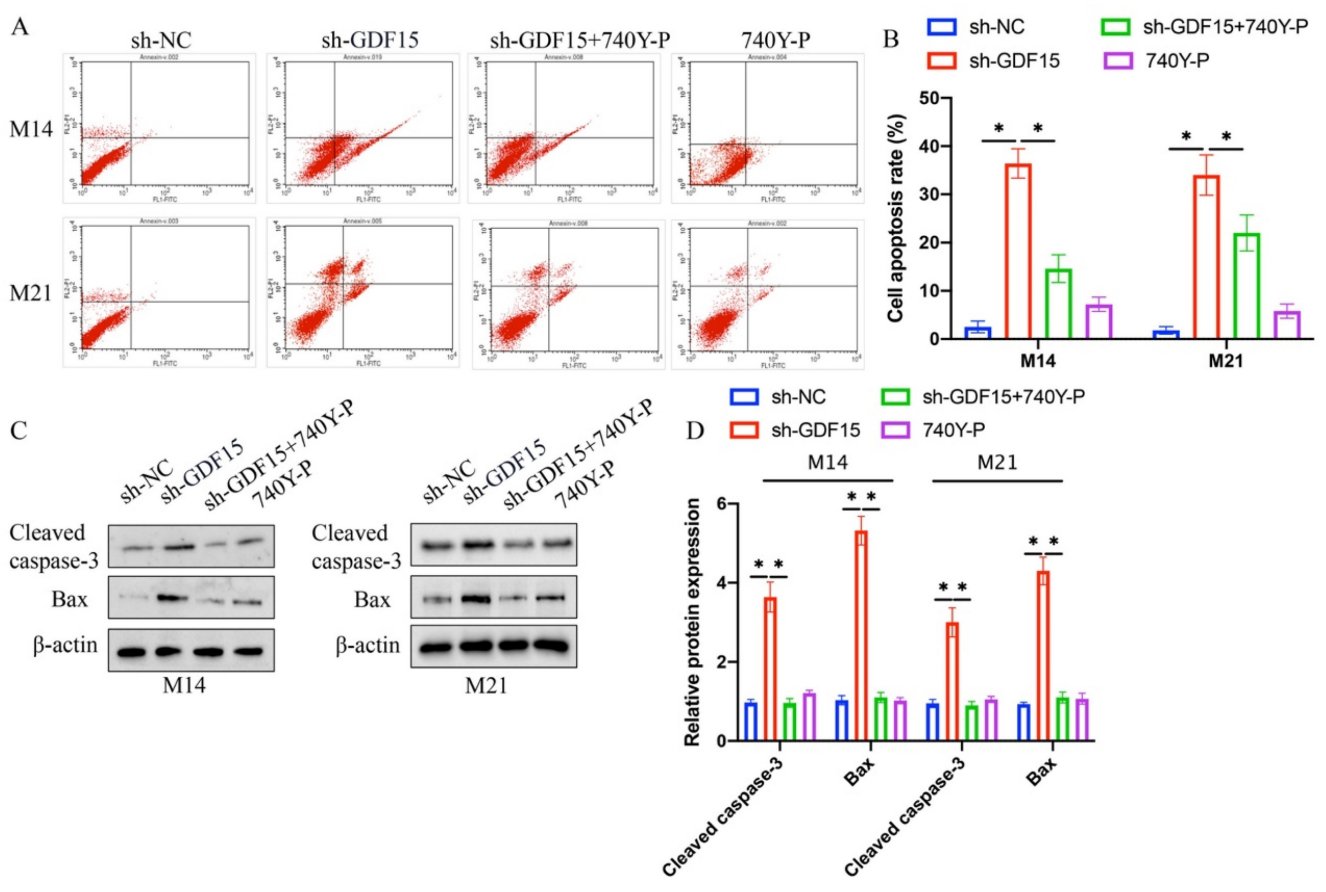
**The increased cell apoptosis caused by sh-GDF15 was inhibited by 740Y-P*.*** (A) The cell apoptosis was measured with flow cytometry; (B) The apoptosis cells were analyzed (n=3); (C) The apoptosis related proteins were measured with western blot; (D) The protein bands were analyzed (n=3). * indicates p<0.05. ANOVA tests was applied in this research for statistical analysis.

**Figure 6:**
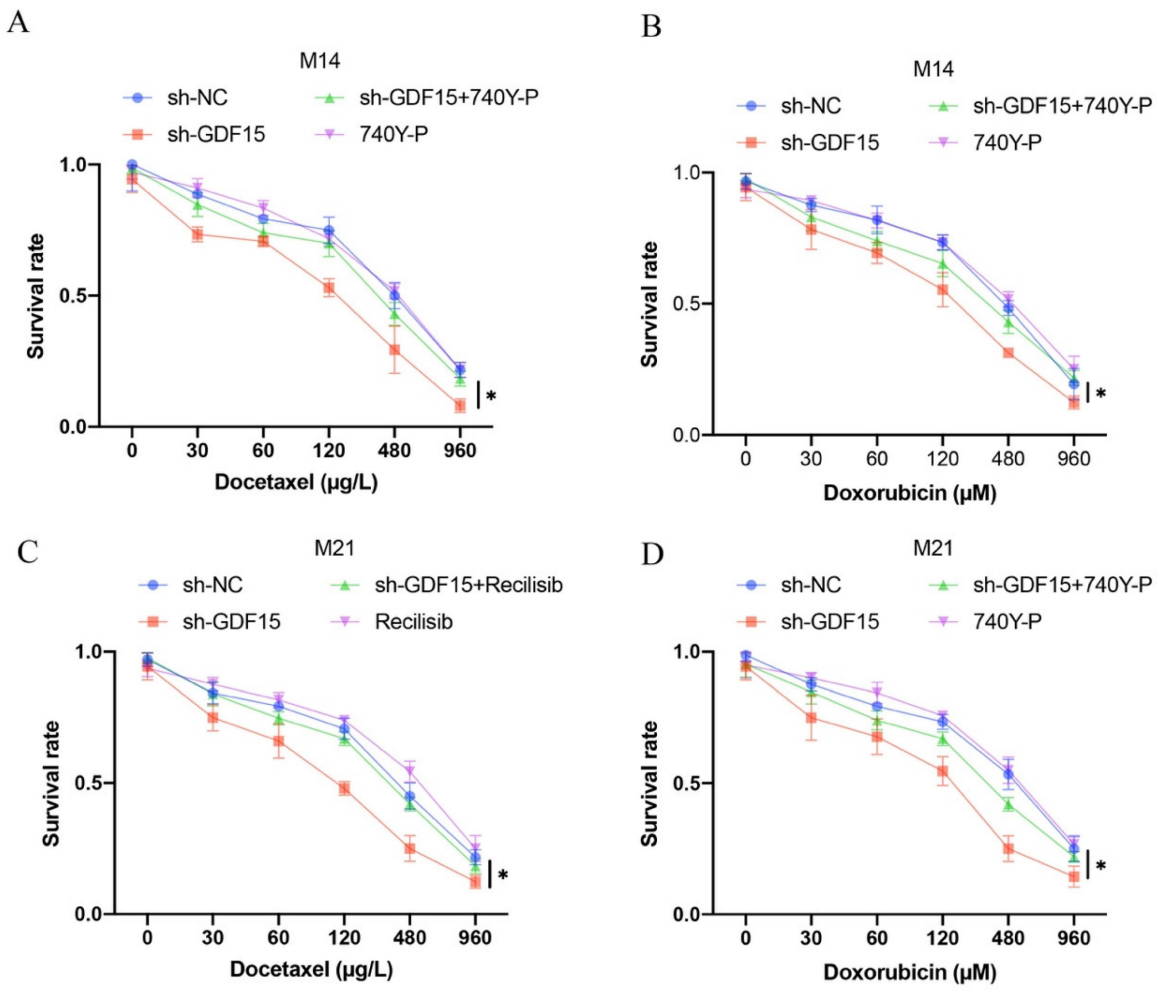
** sh-GDF15 greatly decreased the resistance of M14 and M21 to chemotherapy drugs.** (A) The decreased of M14 resistance to docetaxel by sh-GDF15 was reversed by 740Y-P (n=3); (B) The decreased of M14 resistance to doxorubicin by sh-GDF15 was reversed by 740Y-P (n=3); (C) The decreased of M21 resistance to docetaxel by sh-GDF15 was reversed by 740Y-P (n=3); (D) The decreased of M21 resistance to doxorubicin by sh-GDF15 was reversed by 740Y-P (n=3). * suggests p <0.05. ANOVA tests was applied in this research for statistical analysis.

Increased cell apoptosis induced by sh-GDF15 was diminished following 740Y-P treatment (Figure 5 A-B). Additionally, the elevated levels of pro-apoptotic proteins Bax and cleaved caspase-3 observed post-sh-GDF15 transfection were reduced with 740Y-P treatment (Figure 5 C-D). Furthermore, sh-GDF15 was found to decrease chemotherapy resistance in M14 and M21 cell lines to agents like docetaxel and doxorubicin, an effect that was counteracted by 740Y-P administration (Figure 6 A-D).

### 3.5 740Y-P significantly counterbalanced the impact of sh-GDF15 on the expression of EMT-related proteins in M14 and M21 cell lines

**Figure 7:**
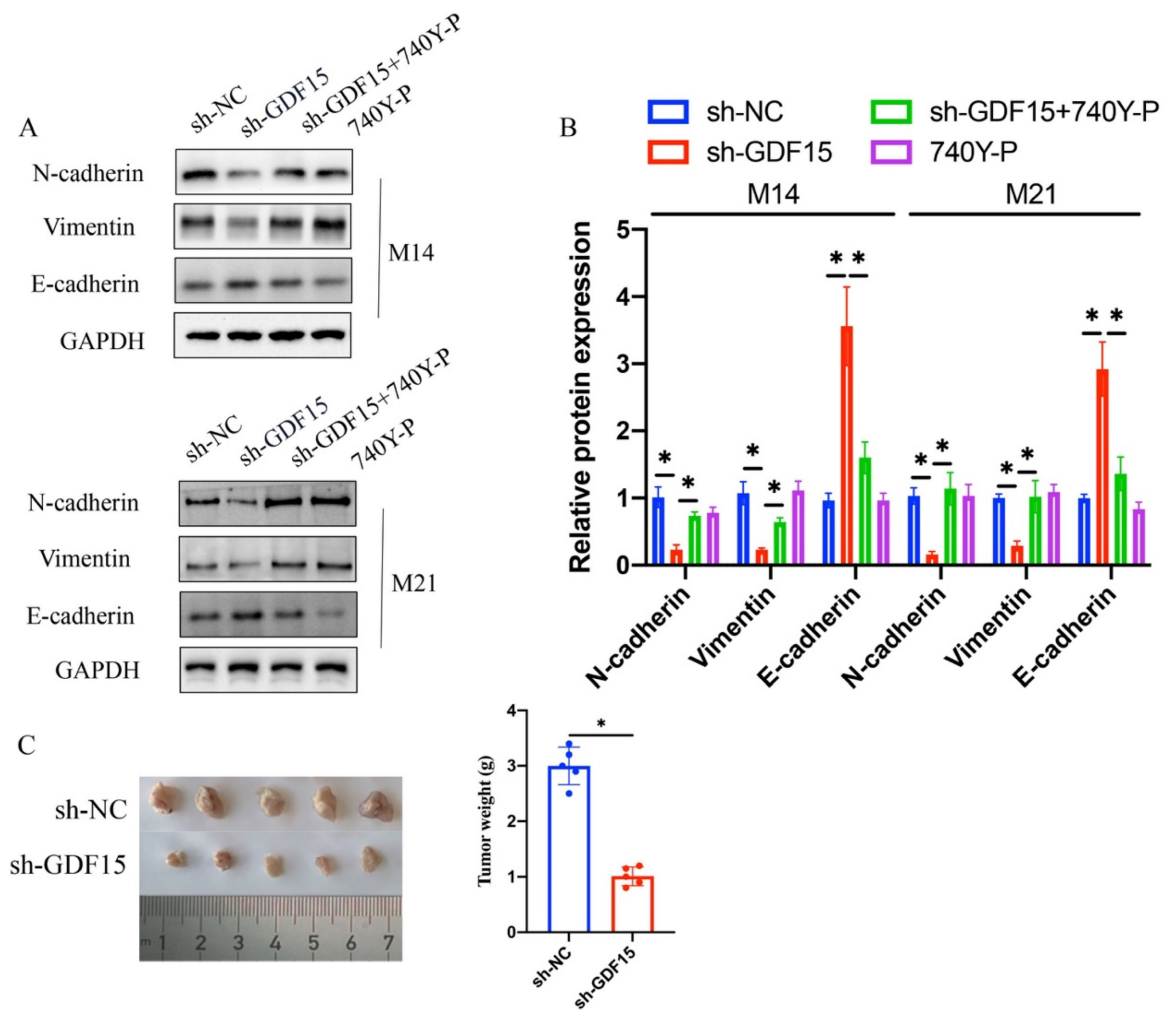
**740Y-P significantly reversed the influence of sh-GDF15 on EMT related proteins expression in M14 and M21 cell lines.** (A) The expression levels of Vimentin, E-cadherin, and N-cadherin were measured with western bolt; (B) The expression levels of Vimentin, E-cadherin, and N-cadherin were analyzed (n=3); (C) Represented tumor growth images were shown and the tumor weight was analyzed (n=5). * means p <0.05. T-test and ANOVA tests were applied in this research for statistical analysis.

Treatment with sh-GDF15 resulted in decreased Vimentin and N-cadherin expression and increased E-cadherin levels in both M14 and M21 cell lines (Figure 7 A-B). However, simultaneous treatment with 740Y-P notably reversed these effects (Figure 7 A-B). These findings indicate that sh-GDF15 might regulate melanoma progression through the PTEN/PI3K/AKT pathway. In vivo validation confirmed sh-GDF15's tumor growth suppression (Figure 7 C), reinforcing our conclusions.

## 4. Discussion

GDF15 is a cytokine that has been found to be upregulated in a variety of tumor types, including breast, colorectal, and lung cancer 17. It has been suggested to play a role in tumor progression and metastasis, as well as in the regulation of cell proliferation and apoptosis 18. Studies have also suggested that GDF15 may be involved in the regulation of angiogenesis, inflammation, and immune responses in different types of tumors 19. It was reported that GDF15 was overexpressed in melanoma cells and was associated with depth of tumor invasion and metastasis 4, which is in line with our findings. However, GDF15 might play different regulatory role in other kinds of tumors. GDF15 could inhibit the growth and bone metastasis of lung adenocarcinoma A549 cells through TGF‑β/Smad signaling pathway 20. Therefore, the regulatory role of GDF15 in tumors is complicated, and needs to be further explored.

The PTEN/PI3K/AKT signaling pathway has been proved to play a key role in the regulation of melanoma 21. Activation of PI3K and AKT can lead to increased cell growth, increased cell migration, and increased resistance to apoptosis, all of which are associated with melanoma progression 22. In addition, PI3K and AKT activation can lead to increased expression of pro-angiogenic factors, which can promote tumor growth and metastasis 23.

GDF15 has been shown to regulate the PTEN/PI3K/AKT signaling pathway in different tumors 24. In the present study, we demonstrated that knockdown of GDF15 remarkably inhibited the PTEN/PI3K/AKT signaling pathway and malignant melanoma. In addition, activation of PTEN/PI3K/AKT signaling pathway by 740Y-P greatly reversed the influence of GDF15 on melanoma, indicating that GDF15 might affect the development of melanoma via targeting PTEN/PI3K/AKT signaling pathway. Previous study indicated that GDF15 could suppress apoptosis in cancer cells by in-activating the caspase cascade, which is in line with our data.

EMT related proteins including N-cadherin, E-cadherin, and Vimentin have been believed to play a vital in the tumor metastasis 25. High expression of N-cadherin is closely linked with tumor metastasis by promoting cell-cell adhesion and migration. Vimentin and E-cadherin have been found to be involved in the regulation of tumor cell motility, invasion and metastasis 26, 27. We proved that sh-GDF15 remarkably suppressed the levels of N-cadherin and Vimentin, but elevated E-cadherin. However, the relegation of GDF15 in EMT related proteins was reversed by treatment with 740Y-P.

There are some limitations in this research. Firstly, what causes overexpression of GDF15 in melanoma cells remain unclear. Secondly, how GDF15 regulate various hallmarks of cancer is not clear. Meanwhile, the mechanisms by which knockdown of GDF15 can affect EMT process is not clear.

## 5. Conclusion

In summary, we proved that sh-GDF15 could suppress the cell proliferation, migration, invasion, and EMT process of M14 and M21 cell lines through targeting PTEN/PI3K/AKT signaling pathway. The influences of sh-GDF15 on cell apoptosis and resistance to chemotherapy were reversed by 740Y-P. Meanwhile, the suppression of tumor growth by sh-GDF15 was validated in vivo level. This research might provide a novel prevention and treatment strategy for melanoma.

## Ethical Approval and Consent to participate

The experimental protocol was approved by Shengli Clinical Medical College of Fujian Medical University, Fujian Provincial Hospital (#2022011).

## Availability of data and material

The data and material used to support the findings of this study are included within the manuscript and supplementary files.

## Author contributions

JZ conceived and designed the experiments; JZ and CC performed the experiments; JZ and CC wrote the paper.

## Abbreviations

GDF15: Growth differentiation factor 15
EMT: Emergency medical technicians

## References

[1] Plaschka M, Benboubker V, Grimont M, Berthet J, Tonon L, Lopez J (2022). ZEB1 transcription factor promotes immune escape in melanoma. J Immunother Cancer.

[2] Chen X, Wang K, Jiang S, Sun H, Che X, Zhang M (2022). eEF2K promotes PD-L1 stabilization through inactivating GSK3beta in melanoma. J Immunother Cancer.

[3] Meng Z, Chen Y, Wu W, Yan B, Zhang L, Chen H (2022). PRRX1 Is a Novel Prognostic Biomarker and Facilitates Tumor Progression Through Epithelial-Mesenchymal Transition in Uveal Melanoma. Front Immunol.

[4] Unal B, Alan S, Bassorgun CI, Karakas AA, Elpek GO, Ciftcioglu MA (2015). The divergent roles of growth differentiation factor-15 (GDF-15) in benign and malignant skin pathologies. Arch Dermatol Res.

[5] Lee J, Jin YJ, Lee MS, Lee H (2022). Macrophage inhibitory cytokine-1 produced by melanoma cells contributes to melanoma tumor growth and metastasis in vivo by enhancing tumor vascularization. Melanoma Res.

[6] Suesskind D, Schatz A, Schnichels S, Coupland SE, Lake SL, Wissinger B (2012). GDF-15: a novel serum marker for metastases in uveal melanoma patients. Graefes Arch Clin Exp Ophthalmol.

[7] Weide B, Schafer T, Martens A, Kuzkina A, Uder L, Noor S (2016). High GDF-15 Serum Levels Independently Correlate with Poorer Overall Survival of Patients with Tumor-Free Stage III and Unresectable Stage IV Melanoma. J Invest Dermatol.

[8] Chen H, Zhou L, Wu X, Li R, Wen J, Sha J (2016). The PI3K/AKT pathway in the pathogenesis of prostate cancer. Front Biosci (Landmark Ed).

[9] Liu HY, Zhang YY, Zhu BL, Feng FZ, Yan H, Zhang HY (2019). miR-21 regulates the proliferation and apoptosis of ovarian cancer cells through PTEN/PI3K/AKT. Eur Rev Med Pharmacol Sci.

[10] Li G, Zhang C, Liang W, Zhang Y, Shen Y, Tian X (2021). Berberine regulates the Notch1/PTEN/PI3K/AKT/mTOR pathway and acts synergistically with 17-AAG and SAHA in SW480 colon cancer cells. Pharm Biol.

[11] Hu M, Zhu S, Xiong S, Xue X, Zhou X (2019). MicroRNAs and the PTEN/PI3K/Akt pathway in gastric cancer (Review). Oncol Rep.

[12] Zhao Y, Li A (2021). miR-19b-3p relieves intervertebral disc degeneration through modulating PTEN/PI3K/Akt/mTOR signaling pathway. Aging (Albany NY).

[13] Hlozkova K, Hermanova I, Safrhansova L, Alquezar-Artieda N, Kuzilkova D, Vavrova A (2022). PTEN/PI3K/Akt pathway alters sensitivity of T-cell acute lymphoblastic leukemia to L-asparaginase. Sci Rep.

[14] Pedri D, Karras P, Landeloos E, Marine JC, Rambow F (2022). Epithelial-to-mesenchymal-like transition events in melanoma. FEBS J.

[15] Pearlman RL, Montes de Oca MK, Pal HC, Afaq F (2017). Potential therapeutic targets of epithelial-mesenchymal transition in melanoma. Cancer Lett.

[16] Chae YK, Chang S, Ko T, Anker J, Agte S, Iams W (2018). Epithelial-mesenchymal transition (EMT) signature is inversely associated with T-cell infiltration in non-small cell lung cancer (NSCLC). Sci Rep.

[17] Lodi RS, Yu B, Xia L, Liu F (2021). Roles and Regulation of Growth differentiation factor-15 in the Immune and tumor microenvironment. Hum Immunol.

[18] Husaini Y, Tsai VW, Manandhar R, Zhang HP, Lee-Ng KKM, Lebhar H (2020). Growth differentiation factor-15 slows the growth of murine prostate cancer by stimulating tumor immunity. PLoS One.

[19] Wischhusen J, Melero I, Fridman WH (2020). Growth/Differentiation Factor-15 (GDF-15): From Biomarker to Novel Targetable Immune Checkpoint. Front Immunol.

[20] Duan L, Pang HL, Chen WJ, Shen WW, Cao PP, Wang SM (2019). The role of GDF15 in bone metastasis of lung adenocarcinoma cells. Oncol Rep.

[21] Chen J, Huang L, Quan J, Xiang D (2021). TRIM14 regulates melanoma malignancy via PTEN/PI3K/AKT and STAT3 pathways. Aging (Albany NY).

[22] Fang M, Zhu D, Luo C, Li C, Zhu C, Ou J (2018). In vitro and in vivo anti-malignant melanoma activity of Alocasia cucullata via modulation of the phosphatase and tensin homolog/phosphoinositide 3-kinase/AKT pathway. J Ethnopharmacol.

[23] Shen Q, Han Y, Wu K, He Y, Jiang X, Liu P (2022). MrgprF acts as a tumor suppressor in cutaneous melanoma by restraining PI3K/Akt signaling. Signal Transduct Target Ther.

[24] Liu Y, Lei J, Ji X, Li C, Chen X, Wang J (2022). Knockdown of growth differentiation factor-15 inhibited nonsmall cell lung cancer through inactivating PTEN/PI3K/AKT signaling pathway. Genes Genomics.

[25] Veloso ES, de Carvalho BA, de Souza Silva FH, Ribeiro TS, Lima BM, Almeida CP (2022). Epithelial-mesenchymal transition inhibition by metformin reduces melanoma lung metastasis in a murine model. Sci Rep.

[26] Cheng R, Gao S, Hu W, Liu Y, Cao Y (2021). Nuclear factor I/B mediates epithelial-mesenchymal transition in human melanoma cells through ZEB1. Oncol Lett.

[27] Wang M, Li S, Wang Y, Cheng H, Su J, Li Q (2020). Gambogenic acid induces ferroptosis in melanoma cells undergoing epithelial-to-mesenchymal transition. Toxicol Appl Pharmacol.

